# Quantification of Minimal Disease by Digital PCR in ALK-Positive Anaplastic Large Cell Lymphoma: A Step towards Risk Stratification in International Trials?

**DOI:** 10.3390/cancers14071703

**Published:** 2022-03-27

**Authors:** Christine Damm-Welk, Federica Lovisa, Giorgia Contarini, Jette Lüdersen, Elisa Carraro, Fabian Knörr, Jan Förster, Martin Zimmermann, Alessandra Sala, Luciana Vinti, Annalisa Tondo, Marta Pillon, Wilhelm Woessmann, Lara Mussolin

**Affiliations:** 1 Pediatric Hematology and Oncology, University Medical Center Hospital Hamburg-Eppendorf, 20246 Hamburg, Germany; c.damm-welk@uke.de (C.D.-W.); jette.luedersen@uke.de (J.L.); f.knoerr@uke.de (F.K.); j.foerster@uke.de (J.F.); w.woessmann@uke.de (W.W.); 2 Maternal and Child Health Department, Padova University, 35128 Padova, Italy; giorgia.contarini@studenti.unipd.it; 3 Istituto di Ricerca Pediatrica Città della Speranza, 35127 Padova, Italy; 4 Pediatric Hematology, Oncology and Stem Cell Transplant Division, Padova University Hospital, 35128 Padova, Italy; elisa.carraro87@gmail.com (E.C.); marta.pillon@unipd.it (M.P.); 5 Mildred Scheel Cancer Career Center HaTriCS4, University Medical Center Hamburg-Eppendorf, 20246 Hamburg, Germany; 6 Pediatric Hematology and Oncology, Hannover Medical School, 30625 Hannover, Germany; zimmermann.martin@mh-hannover.de; 7 Department of Paediatric Hematology Oncology, San Gerardo Hospital, University of Milano-Bicocca MBBM Foundation, 20900 Monza, Italy; ale.sala@asst-monza.it; 8 Department of Paediatric Haematology/Oncology, Cell and Gene Therapy, Bambino Gesù Children’s Hospital, IRCCS, 00165 Rome, Italy; luciana.vinti@opbg.net; 9 Department of Pediatric Oncology, Meyer Children’s University Hospital, 50139 Firenze, Italy; annalisa.tondo@meyer.it

**Keywords:** ALCL, childhood, droplet digital PCR, minimal disseminated disease, minimal residual disease

## Abstract

**Simple Summary:**

Detection of minimal disease in blood or bone marrow is associated with high relapse risk in children with anaplastic large cell lymphoma (ALCL). The persistence of minimal residual disease after one course of chemotherapy indicates a relapse risk of 80%. While quantification of minimal disease might further improve the identification of high-risk patients, the assays used for quantification currently are not transferable between multiple laboratories. We aimed to test a digital PCR method (dPCR) for comparison of minimal disease quantification between two laboratories and the usefulness of quantification for risk stratification of children with ALCL. Quantification of minimal disease by dPCR was concordant between laboratories and allowed identification of patients at very high risk for relapse. Qualitative detection of minimal residual disease after one course of chemotherapy sufficed to identify children at the highest risk of treatment failure. International dissemination of this assay will allow patient selection for new targeted treatment approaches.

**Abstract:**

Minimal disseminated and residual disease (MDD/MRD) analyzed by qualitative PCR for *NPM-ALK* fusion transcripts are validated prognostic factors in pediatric ALK-positive anaplastic large cell lymphoma (ALCL). Although potentially promising, MDD quantification by quantitative real-time PCR in international trials is technically challenging. Quantification of early MRD might further improve risk stratification. We aimed to assess droplet digital PCR for quantification of minimal disease in an inter-laboratory setting in a large cohort of 208 uniformly treated ALCL patients. Inter-laboratory quality control showed high concordance. Using a previously described cut-off of 30 copies *NPM-ALK/*10^4^ copies *ABL1* (NCN) in bone marrow and peripheral blood, MDD quantification allowed identification of very high-risk patients (5-year PFS% 34 ± 5 for patients with ≥30 NCN compared to 74 ± 6 and 76 ± 5 for patients with negative or <30 NCN, respectively, *p* < 0.0001). While MRD positivity was confirmed as a prognostic marker for the detection of very high-risk patients in this large study, quantification of MRD fusion transcripts did not improve stratification. PFS% was 80 ± 5 and 73 ± 6 for MDD- and MRD-negative patients, respectively, versus 35 ± 10 and 16 ± 8 for MRD-positive patients with <30 and ≥30 NCN, *p* < 0.0001. Our results suggest that MDD quantification by dPCR enables improved patient stratification in international clinical studies and patient selection for early clinical trials already at diagnosis.

## 1. Introduction

Anaplastic Lymphoma Kinase (ALK)-positive anaplastic large cell lymphoma (ALCL) is characterized by oncogenic chromosomal translocations involving the *ALK* gene localized on chromosome 2p23. The t(2;5)(p23;q35) leading to the fusion gene *NPM-ALK* accounts for more than 80% of ALK-positive ALCL in children [1,2].

Risk factor analyses established the detection of minimal disseminated disease (MDD) in bone marrow (BM) or peripheral blood (PB) by qualitative RT-PCR for *NPM-ALK* fusion transcripts, small-cell or lymphohistiocytic patterns and low anti-ALK antibody titers as independently associated with treatment failure [3,4]. Detection of minimal residual disease (MRD) after one course of chemotherapy conferred a very high risk of relapse [5,6]. MDD has been validated as prognostic factor in several independent clinical trials [4,5,7,8,9,10,11,12].

Measurement of qualitative MDD in BM and PB has evolved to a routine staging procedure for patients with ALK-positive ALCL [13,14]. MRD assessment in PB after one course of chemotherapy as well as further during therapy in positive patients belongs to routine restaging, as well [14,15]. These parameters are now used as inclusion criteria or for patient stratification in clinical trials (ALCL-VBL EudraCT: 2017-002935-40, Briga-Ped: NCT04925609).

The Berlin-Frankfurt-Münster (BFM) study group previously demonstrated the potential of MDD quantification by quantitative real-time PCR (qPCR) in children treated with ALCL99 chemotherapy. Children with more than 10 copies *NPM-ALK*/10^4^ *ABL1* (normalized copy numbers, NCN) in BM or PB showed a significantly higher risk of treatment failure compared to those who were MDD-negative or “low positive” (≤10 NCN) [9,10]. However, while quantification of MDD using this same method and the cut-off of 10 NCN was associated with a high risk of relapse in three other cohorts (an Associazione Italiana di Ematologia e Oncologia Pediatrica (AIEOP) and a Japanese cohort treated with identical therapy and the COG-study ANHL12P1 combining ALCL99 with brentuximab vedotin), the reported relative cohort sizes and relapse risks of patients with MDD >10 NCN grossly differed between the four studies [9,11,12,16]. Since qPCR relies on the use of plasmid standard curves for quantification of transcripts, even slight differences in copy numbers between the standards for *NPM-ALK* and/or *ABL1* used in different laboratories may result in large differences in NCN. Furthermore, quantification of MDD in ALK-positive ALCL is necessary at very low copy numbers reaching the lowest dilution of the standard curve so that differences between laboratories are almost unavoidable when using qPCR. Therefore, the published studies on quantitative MDD using qPCR in ALK-positive ALCL have always selected one central laboratory for the analyses. The necessary cut-off at low copy numbers poses a huge challenge for harmonization of the method between laboratories even if a centrally produced standard curve would be chosen.

Quantification by digital droplet PCR (dPCR) is based on the principle of limiting dilution. The target molecules are distributed to many partitions, so that, theoretically, a single target molecule can be amplified in each partition [17]. Using Poisson statistics, the number of PCR positive and negative partitions enables the absolute quantification of the initial target molecules without the need for a standard curve [18,19]. The simplicity of the method, as well as high precision to detect rare events, qualifies dPCR for MDD and MRD analysis, especially to reach comparability between laboratories at low-level minimal disease. Recently, dPCR has been proposed as a tool for reproducible quantification of fusion gene transcripts like *BCR-ABL1* in leukemias [20] and also *NPM-ALK* transcripts in children with ALCL [10].

MRD detection by qualitative RT-PCR for *NPM-ALK* before the second course of chemotherapy has enabled the identification of patients at the highest risk of relapse of 80% in a collaborative AIEOP-BFM analysis which could be replicated and validated by the French group [5,6]. However, it is currently unknown whether MRD quantification might further improve patient stratification.

In the present cooperative study between the AIEOP and the BFM national reference laboratories, we investigated the inter-laboratory concordance and prognostic value of MDD and MRD quantification by dPCR in a large cohort of *NPM-ALK*-positive ALCL patients, uniformly treated with ALCL99-type chemotherapy.

## 2. Materials and Methods

### 2.1. Patients

Children and adolescents with ALK-positive ALCL, confirmed by central histopathology and enrolled in the ALCL99 trial or the NHL-BFM Registry 2012 by the AIEOP and BFM study groups, between April 2004 and April 2020, were included in this retrospective study. Eligibility criteria were genetic or immune-histological confirmation of *NPM-ALK* positivity [4,10], chemotherapy according to ALCL99 and the availability of qualitative MDD results in BM and/or PB. Patients with completely resected stage I disease or isolated skin lesions were excluded since they received shorter or no chemotherapy. Overall, 208 patients fulfilled the criteria (84 AIEOP; 124 BFM).

Both studies were approved by their respective Institutional Review Board and Ethics Committee. The patients, parents or legal guardians provided written informed consent to the studies including MDD and MRD analyses.

### 2.2. Complementary DNA Synthesis and Qualitative Polymerase Chain Reaction

Total RNA was obtained from BM/PB mononuclear cells by using TRIzol Reagent (ThermoFisher Scientific), following the manufacturer’s instructions. RNA reverse transcription (RT) into complementary DNA (cDNA) and subsequent qualitative PCR for *NPM-ALK* were performed as previously reported [7]. Briefly, 1 µg of total RNA was reversed transcribed using 200 U SuperScript II reverse transcriptase (ThermoFisher Scientific) and random hexamers, following the manufacturer’s recommendations.

### 2.3. Digital Polymerase Chain Reaction Assay

Monoplex or duplex dPCR assays for NPM-ALK and ABL1 (reference gene) amplification were performed. Primer and probes were as follows: NPM-ALK: 5′-CAGTGCATATTAGTGGACAGCACTTAG-3′, 5′-TGATGGTCGAGGTGCGGA-3′ and the probe 5′-CACCAGGAGCTGCAAGCCATGCA-3′; ABL1: 5′-CAACACTGCTTCTGATGGCAA 3′, 5′-CGGCCACCGTTGAATGAT-3′ and the probe 5′- CAACACCCTGGCCGAGTTGGTTCAT-3’ with 5′ 6-FAM™/HEX™ (monoplex/duplex) as reporter dyes and ZEN™ and 3′IowaBlack^®^FQ double quencher dyes for the probes (IDT, Leuven, Belgium). dPCR was performed in a reaction volume of 20 µL using 1X dPCR^TM^ supermix for probes no dUTP (Bio-Rad, Munich, Germany), 900 nM of each primer, 250 nM of probes and 1 µL of the RT product. Patients’ samples were analysed in triplicate. cDNA from the following cell lines were used as negative and positive controls: KM-H2 (Hodgkin lymphoma), HL-60 (acute myeloid leukaemia) or DG-75 (Burkitt lymphoma), Karpas 299 and SR-786 (NPM-ALK positive ALCL). The cDNA of positive controls was diluted at least 1:10 in cDNA from ALK-negative cell lines to ensure the occurrence of negative droplets. All cell lines were received from the German Collection of Microorganisms and Cell Cultures (DSMZ, Braunschweig, Germany). Positive, negative, and no-template controls were analysed in duplicates. Droplets were generated with the QX-200 droplet generator (Bio-Rad). End-point PCR was then performed as previously described [10]. Droplets were measured with the QX200 droplet reader (Bio-Rad, Munich, Germany) and analysed with the QuantaSoft Pro analysis software V 1.7.4.0917 (Bio-Rad). Only replicates with a minimum of 10,000 droplets and ≥1000 copies of the reference gene ABL1 were included in the analysis. A single threshold to discriminate between positive and negative droplets was established manually for all the samples in the analysis and set above the background signal. Samples were defined as positive if at least a total of three droplets were positive, regardless of the number of positive replicates; samples were defined as negative if ≤one positive droplet was observed; samples were defined as positive not quantifiable (PNQ) when a total of two droplets were positive. NPM-ALK copy number was normalized to 10,000 copies of ABL1 to calculate the normalized copy number (NCN) of each positive sample. Quantitative MDD analyses from BFM patients diagnosed between 2004 and 2016 have been previously published [10].

Sequences for the NPM-ALK/ABL1 synthetic double-stranded DNA fragments (gBlocks^®^, Integrated DNA Technologies, Leuven, Belgium) were previously published [21]. Dilutions from 2 × 10^5^ molecules/µL to 2 molecules/µL were performed in 50 µg/mL *E. coli* tRNA (Roche, Mannheim, Germany).

### 2.4. Statistical Analysis

Descriptive statistics were used to summarize the collected data. Survival analyses were performed according to the Kaplan–Meier method and the survival functions obtained were compared using the log-rank test [22]. Progression-free survival (PFS) was calculated from the date of diagnosis to the date of the first event (relapse, refractory disease or disease progression) or to the date of the last follow-up. Overall survival (OS) was calculated from the date of diagnosis to the date of death for any reason or to the last follow-up whichever occurred first. All *p*-values are two-sided, with a type I error rate fixed at 0.05. Comparisons of dPCR results were performed with Spearman correlations. Associations between patient characteristics were analysed by the Chi-square or Fisher’s exact tests.

Data analyses were performed by using SAS statistical analysis software (SAS-PC, version 9.4, SAS Institute, Cary, NC, USA) and GraphPad Prism 7.0 (San Diego, CA, USA).

## 3. Results

### 3.1. Optimization of the dPCR Protocol and Inter-Laboratory Concordance

To simplify MDD analysis for international studies, a duplex dPCR approach was tested to allow quantification of NPM-ALK and ABL1 copy numbers in a single reaction set. NCN in 26 BM and 26 paired PB samples measured by monoplex and duplex dPCR were highly concordant (Spearman ρ = 0.92 and 0.99 in BM and PB, respectively) (Supplementary Figure S1).

An inter-laboratory comparison was performed with three different quality control sets:

(1) 10-fold serial dilutions (range 10^−1^ to 10^−5^) of cDNA of the ALK-positive cell line SR-786 in cDNA from the ALK-negative cell line DG-75. In addition, the DG-75 cDNA was used as a negative control.
(2) Thirty-three cDNAs from clinical samples with positive, low positive or negative MDD.
(3) A synthetic NPM-ALK/ABL1 gBlock^®^ fragment that was diluted to 2, 20, 200, 2000, 20,000, and 200,000 calculated target molecules.

**Figure 1:**
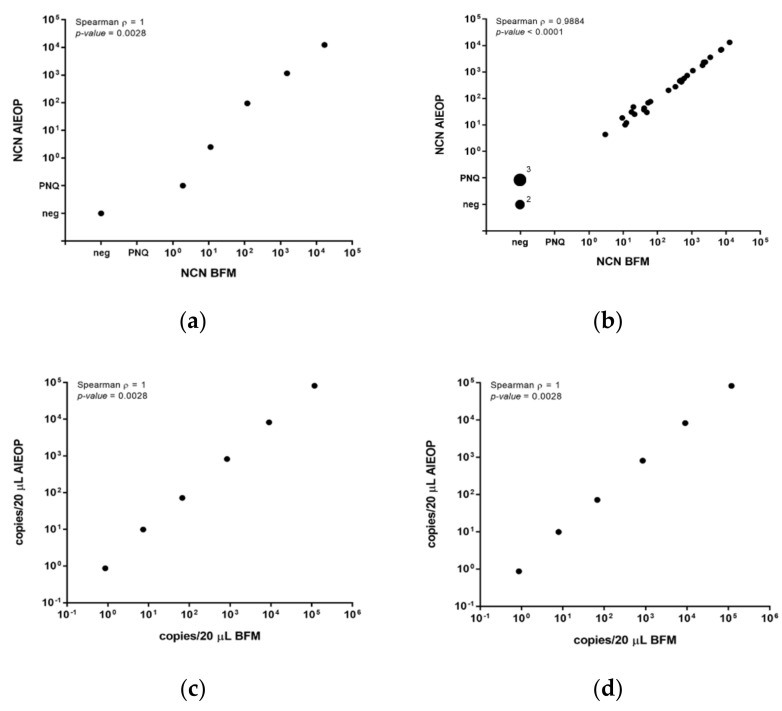
Quality control for MDD/MRD quantification by qPCR in an inter-laboratory setting. Comparison of normalized copy numbers (NCN) NPM-ALK (copies NPM-ALK/10^4^ copies ABL1) measured by AIEOP and BFM laboratories in quality control samples represented by five serial dilutions of cDNAs from of the NPM-ALK-positive cell line SR-786 in DG-75 and a negative control (**a**) and by 33 clinical samples from *NPM-ALK*-positive ALCL patients (**b**). Comparison of NPM-ALK (**c**) and ABL1 (**d**) copies/20 µL in six serial dilutions (200,000 to 2 molecules) of gBlock^®^ Gene Fragments with NPM-ALK and ABL1 sequences.

The NCN results of all three quality control sets were highly concordant between the laboratories, with a Spearman correlation coefficient ρ = 1.0 for the cell line dilutions (Figure 1a), ρ = 0.99 for the clinical samples (Figure 1b) and ρ = 1.0 for the gBlock^®^ fragment, respectively (Figure 1c,d).

### 3.2. Patient Characteristics

The clinical and biological characteristics of the study population according to MDD are summarized in Table 1.

The study cohort included 208 pediatric *NPM-ALK*-positive ALCL patients, 135 males and 73 females. The median age at diagnosis was 12.1 years (range 0.25–18 years). Among them, 80 patients experienced disease progression or relapse; 12/80 died of progressive disease (*n* = 8) or treatment related mortality (*n* = 4). One additional patient died of initial tumor complications (uncontrolled cytokine storm with hemophagocytic lymphohistiocytosis (HLH) despite intensive chemotherapy and HLH-therapy). The median follow-up was 5.3 years (range 0.43–17.5). Central nervous system involvement at diagnosis was detected in three patients.

The five-year OS% and PFS% (±SE%) for the whole study cohort were 94 ± 2 and 61 ± 3, respectively.

### 3.3. Prognostic Significance of Quantitative Minimal Disseminated Disease

MDD could be quantified in 204 patients. For 138/204, both BM and PB were available for MDD analysis by dPCR. For 53/204 and 13/204 patients MDD has been assessed only on BM or PB samples, respectively.

**Figure 2:**
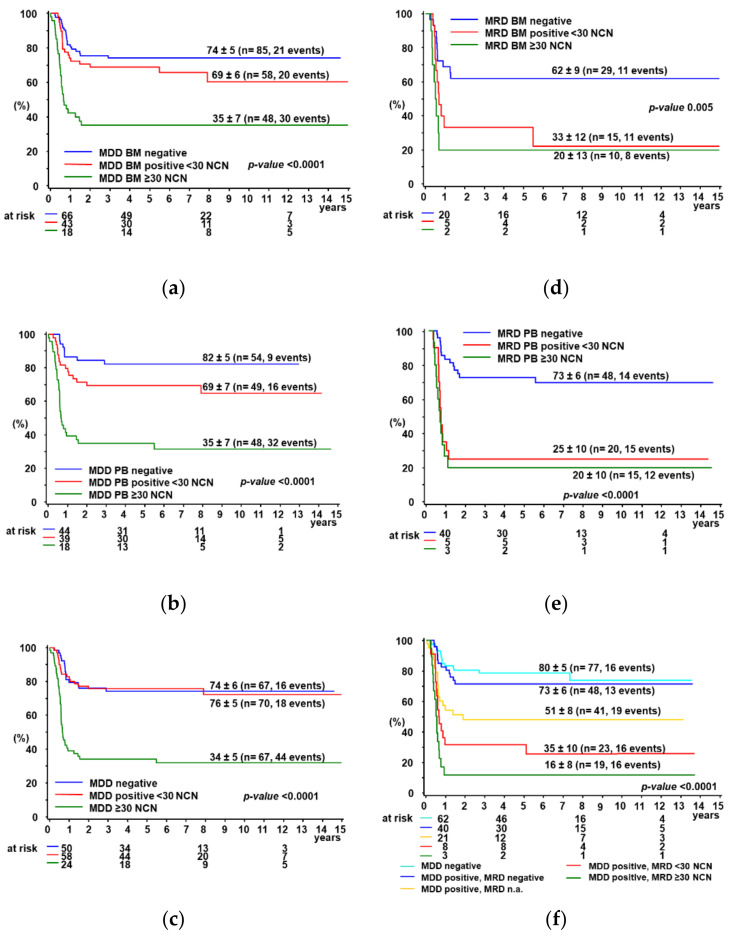
5-year PFS% according to MDD (negative, positive < 30 NCN or ≥30 NCN) in BM (**a**), PB (**b**), combined (**c**) and according to MRD (negative, positive < 30 NCN or ≥30 NCN) in BM (**d**), PB (**e**) and combined (**f**). Definition for combined groups (**c**,**f**): MDD/MRD negative: both BM and PB were negative or one of them if only one was available; MDD/MRD positive: the highest measured NCN was chosen between BM and PB determinations or one of them, if only one was available.

MDD was positive in 82/191 BM samples, 24/191 were positive but not quantifiable (PNQ) and 85/191 were negative. To evaluate the prognostic significance of MDD quantification by dPCR, we stratified patients according to different cut-off, choosing at the end the previously published cut-off of 30 NCN [10], showing the best separation between patients with or without progression/relapse. Patients were grouped in high-positive (≥30 NCN), low-positive (<30 NCN) or negative. The five-year PFS% (±SE%) was 35 ± 7 for high-positive patients, 69 ± 6 for low-positive and 74 ± 5 for negative patients (Figure 2a, *p* < 0.0001). Notably, the PFS was not significantly different between patients with negative and low-positive MDD (*p* = 0.22).

As for MDD in PB, 75/151 samples were positive, 22/151 were PNQ and 54/151 were negative. Using the same criteria for patients’ stratification as for BM, the five-year PFS% (±SE%) was significantly lower for high-positive (35 ± 7) compared to low-positive (69 ± 7) and negative (82 ± 5) patients (Figure 2b, *p* < 0.0001, *p* = 0.08 for low-positive versus negative).

Quantitative MDD results in BM and PB were highly concordant (Supplementary Figure S2a, Spearman ρ = 0.80).

In patients with an MDD ≥ 30 NCN, either in BM or PB, the PFS% was 34 ± 5, significantly lower compared to patients with positive MDD < 30 NCN (PFS% 76 ± 5) or with negative MDD (PFS% 74 ± 6) in both BM and PB or one of them, if only one was available (Figure 2c, *p* < 0.0001). MDD results in paired BM and PB samples are reported in Supplementary Table S1. Overall survival curves according to MDD in BM and/or PB are reported in Supplementary Figure S3, whereas the prognostic impact of qualitative MDD results measured in BM and PB is shown in Supplementary Figure S4.

### 3.4. Prognostic Significance of Quantitative Minimal Residual Disease

BM and/or PB samples before the second course of chemotherapy were available from 90 MDD-positive patients for MRD quantification by dPCR. Both BM and PB were analyzed in 47/90 patients, whereas for 7/90 and 36/90 patients MRD has been assessed on BM or PB samples only, respectively. Quantitative MRD results in BM and PB were highly concordant (Supplementary Figure S2b, Spearman ρ = 0.87).

Using a threshold of 30 NCN *NPM-ALK*, no significant difference in PFS% was observed between patients with high-positive or low-positive MRD in BM and PB, respectively (Figure 2d,e). Patients with any MRD positivity by dPCR in either BM or PB had a significantly lower PFS% compared to MRD negative and MDD negative patients (Figure 2f. MRD results in paired BM and PB samples are reported in Supplementary Table S2. Overall survival curves according to MDD in BM and/or PB are reported in Supplementary Figure S3, whereas the prognostic impact of qualitative MDD results measured in BM and PB is shown in Supplementary Figure S4.

## 4. Discussion

Quantification of minimal disease has been established as the standard for MRD determination in leukemias [23]. qPCR-based assays for Ig/TCR rearrangements with rigorous inter-laboratory control reach a high concordance of quantification between laboratories down to a level of 10^−4^ [24]. Sufficient concordance for qPCR-based MRD quantification using fusion gene transcripts like *BCR-ABL1* between laboratories could only be reached by applying stringent quality control and centrally distributed plasmid standard curves [20].

In lymphomas, the amount of MDD is a prerequisite to judge MRD results and quantification of fusion gene transcripts has to be normalized against a control gene standard. Furthermore, minimal disease is often limited to a few tumor cells, so that, especially for ALCL, quantification is necessary at a very low level, near the limit of detection and quantification of the qPCR-based method. The current experience on MDD quantification by qPCR in children with *NPM-ALK*-positive ALCL underlines that qPCR results cannot be compared between laboratories [9,10,11,12,16]. Although detection of MDD was associated with a higher risk of relapse in all studies, the relative size of the patient group with >10 NCN and the increase in relapse risk of these children compared to those with low or no copies detectable varied broadly.

A dPCR-based approach allows a more precise detection, especially of rare events, without needing standard curve calibration, making the method attractive for inter-laboratory minimal disease detection in lymphomas [10,21,25,26]. A collaborative inter-laboratory work by the mantle cell lymphoma network demonstrated that dPCR resulted in more solid quantification of samples with positivity between 10^−4^ and 10^−5^ compared to qPCR [27]. For Philadelphia positive ALL patients, quantification of low numbers of *BCR-ABL1* fusion gene transcripts by dPCR reached a higher accuracy and reproducibility compared to qPCR [20].

Our results show that dPCR reaches a high inter-laboratory reproducibility for minimal disease quantification in *NPM-ALK*-positive ALCL. Together with the introduction of a duplex dPCR-approach for the target and control transcript, the method proved very suitable for minimal disease quantification for ALCL patients in an inter-laboratory and international setting. Using the previously suggested cut-off of 30 NCN, we confirmed the possibility to identify a very-high risk group of ALCL patients by MDD quantification with dPCR [10]. A patient group of only 25–30% of patients with a risk of relapse of 65% could be separated in an international setting in a large patient cohort. Compared to qualitative RT-PCR for *NPM-ALK*, which is positive in 50–60% of patients with a relapse risk of 50% [4,5,7,8,9,10], quantification by dPCR allowed defining the group of high-risk patients more accurately.

Currently, qualitative RT-PCR is used for quality-controlled MDD detection for patients’ stratification in international clinical trials. Although quality control reached a very high level of concordance for RT-PCR, borderline cases pose a challenge for the interpretation of results. Our findings suggest that quantification by dPCR might enable overcoming this limitation.

Quantification of MDD correlated in samples from BM and PB, as it has been shown in previous studies with lower patient numbers from single laboratories [9,10]. In line with previous findings, we could confirm that copy numbers in PB were slightly higher compared to BM, further underlining that minimal disease in ALCL can be regarded as circulating tumor cells and not marrow metastasis. Since initial staging in pediatric lymphomas includes BM cytology and histology, both PB and BM are available for MDD quantification. Further inter-laboratory analyses and discussions in the study groups will clarify whether quantification from PB or BM should be primarily used for patient stratification.

Quantification of early MRD by dPCR did not enable a more precise risk group definition compared to qualitative detection of MRD by RT-PCR in our study. In line with previous studies, the sole detection of MRD after only one course of chemotherapy conferred a very high risk of treatment failure of more than 70% [5,6]. Within this patient cohort, children with low or high copy numbers measured by dPCR showed a comparable relapse risk, whereas the risk of failure of children without detectable MRD after one course of chemotherapy was as low as the one of children with negative MDD. Although quantification of MRD did not further improve prediction of relapse risk when used early during initial therapy, the high precision of MRD measurement by dPCR will improve inter-laboratory comparability of longitudinal MRD monitoring in relapsed patients, e.g., those on ALK inhibitor therapy, in whom often a slow but steady decline in copy number is observed, that is difficult to assess with qPCR. Due to the relative quantification by qPCR and the associated variability in measurement, therapeutic significance of a slight decrease in copy number is much more difficult to assess. The determination of a slow decrease in *NPM-ALK* copy numbers will therefore be much more accurate by using dPCR in long-term monitoring.

Our study also has some limitations. First of all, multiple QC rounds need to be performed involving multiple central laboratories before translating this assay to international clinical studies, both to confirm the suggested cut-off of 30 NCN and to clarify which is the best clinical sample (BM or PB) to be analyzed for initial stratification. Moreover, despite the fact that our assay can be applied to 95% of ALCL cases, expressing the *NPM-ALK* fusion transcript, patients bearing variant translocations, such as *TPM3-ALK* or *ATIC-ALK* cannot be analyzed using this *NPM-ALK* specific assay. Recently, a dPCR approach using a 3′*ALK* universal probe has been proposed, which can be applied to the remaining cases [21].

## 5. Conclusions

In conclusion, we could establish dPCR as a tool for minimal disease quantification for patients with *NPM-ALK*-positive ALCL in an international setting. International inter-laboratory quality control of minimal disease quantification by dPCR is going to be set up within the European Inter-Group for Childhood NHL network of national reference laboratories. For MDD, the cut-off of 30 NCN could be confirmed to identify patients at a very high risk of failure. The sole detection of MRD after only one course of chemotherapy suffices to define patients with a relapse risk of almost 80%. However, MRD quantification offers the possibility to follow the disease course of an individual patient more precisely.

## Figures and Tables

**Table 1:** Patient characteristics’ of 204 NPM-ALK-positive ALCL-patients according to minimal disseminated disease (MDD).

		Quantitative MDD by dPCR											
All Patients *n* = 208 *		In Bone Marrow *n* = 191				In Peripheral Blood *n* = 151				Combined # *n* = 204			
		Neg	<30 NCN	≥30 NCN	*p*	Neg	<30 NCN	≥30 NCN	*p*	Neg	<30 NCN	≥30 NCN	*p*
		85	58	48		54	49	48		67	70	67	
**Gender, *n* (%)**					0.79				0.81				0.91
Male	135	54 (64%)	40 (69%)	32 (67%)		36 (67%)	31 (63%)	29 (60%)		44 (66%)	46 (66%)	42 (63%)	
Female	73	31 (36%)	18 (31%)	16 (33%)		18 (33%)	18 (37%)	19 (40%)		23 (34%)	24 (34%)	25 (37%)	
**Age, *n* (%)**					0.63				0.15				0.06
<12.1 years	104	41 (48%)	28 (48%)	27 (56%)		25 (46%)	22 (45%)	30 (63%)		32 (48%)	29 (41%)	41 (61%)	
≥12.1 years	104	44 (52%)	30 (52%)	21 (44%)		29 (54%)	27 (55%)	18 (37%)		35 (52%)	41 (59%)	26 (39%)	
**Stage, *n* (%)**					<0.0001				0.0005				<0.0001
1–2	45	29 (34%)	12 (21%)	1 (2%)		18 (33%)	10 (20%)	2 (4%)		26 (39%)	15 (21%)	3 (4%)	
3–4	153	55 (65%)	40 (69%)	44 (92%)		34 (63%)	37 (76%)	44 (92%)		40 (60%)	49 (70%)	61 (91%)	
n.a.	10	1 (1%)	6 (10%)	3 (6%)		2 (4%)	2 (4%)	2 (4%)		1 (1%)	6 (9%)	3 (5%)	
**BM, *n* (%)**					0.001				0.0002				0.0003
Negative	180	83 (98%)	48 (83%)	35 (73%)		52 (96%)	45 (92%)	32 (67%)		66 (99%)	61 (87%)	49 (73%)	
Positive	13	1 (1%)	2 (3%)	8 (17%)		0	2 (4%)	9 (19%)		0	3 (4%)	10 (15%)	
n.a.	15	1 (1%)	8 (14%)	5 (10%)		2 (4%)	2 (4%)	7 (14%)		1 (1%)	6 (9%)	8 (12%)	
**CNS, *n* (%)**					0.06				0.02				0.03
Negative	189	83 (98%)	51 (88%)	40 (83%)		52 (96%)	46 (94%)	38 (79%)		66 (99%)	63 (90%)	56 (84%)	
Positive	3	0	0	2 (4%)		0	0	3 (6%)		0	0	3 (4%)	
n.a.	16	2 (2%)	7 (12%)	6 (13%)		2 (4%)	3 (6%)	7 (15%)		1 (1%)	7 (10%)	8 (12%)	
**Histology *n* (%)**					0.0005				0.13				0.002
Non-common	75	24 (28%)	22 (28%)	23 (48%)		14 (26%)	16 (33%)	19 (40%)		20 (30%)	23 (33%)	32 (48%)	
Common	94	52 (61%)	23 (40%)	9 (19%)		27 (50%)	27 (55%)	15 (31%)		39 (58%)	35 (50%)	16 (24%)	
n.a.	39	9 (11%)	13 (22%)	16 (33%)		13 (24%)	6 (12%)	14 (29%)		8 (12%)	12 (17%)	19 (28%)	

n.a., not available; * four patients with positive MDD by qualitative RT-PCR could not be quantified by dPCR but had material for MRD quantification; # highest value of BM and PB counts for grouping.

## Data Availability

Data is contained within the article or supplementary material.

## Supplementary Materials

The following supporting information can be downloaded at: https://www.mdpi.com/article/10.3390/cancers14071703/s1, Figure S1: Comparison of monoplex and multiplex dPCR assay for MDD/MRD quantification. Figure S2: Comparison of MDD and MRD quantification in BM and PB. Figure S3: 5-year OS% according to MDD results (negative, positive < 30 NCN or ≥30 NCN) in BM (a), PB (b), combined (c)., Figure S4: 5-year PFS% according to qualitative MDD results (negative or positive) in BM (a) or PB (b). Figure S5: 5-year OS% according to MRD results (negative, positive < 30 NCN or ≥30 NCN) in BM (**a**), PB (**b**) and combined (**c**), Figure S6: 5-year PFS% according to qualitative MDD and MRD results (negative or positive) in BM and/or PB, Table S1: MDD results in paired BM and PB samples or one of them, if only one was available, Table S2: MRD results in paired BM and PB samples or one of them, if only one was available.
