# Supplementary material for: Quantification of Minimal Disease by Digital PCR in ALK-Positive Anaplastic Large Cell Lymphoma: A Step towards Risk Stratification in International Trials?
Source: Cancers (Basel). 2022 Mar 27;14(7):1703. doi: 10.3390/cancers14071703 (PMC8996924; doi:10.3390/cancers14071703)
Supplement: Supplementary file 1 [file cancers-14-01703-s001.zip › cancers-1659358-supplementary.pdf]

Article

Quantification of Minimal Disease by Digital PCR in ALK-Positive Anaplastic Large Cell Lymphoma: A Step towards Risk Stratification in International Trials?

Christine Damm-Welk, Federica Lovisa, Giorgia Contarini, Jette Lüdersen, Elisa Carraro, Fabian Knörr, Jan Förster, Martin Zimmermann, Alessandra Sala, Luciana Vinti, Annalisa Tondo, Marta Pillon, Wilhelm Woessmann and Lara Mussolin

Supplementary Materials

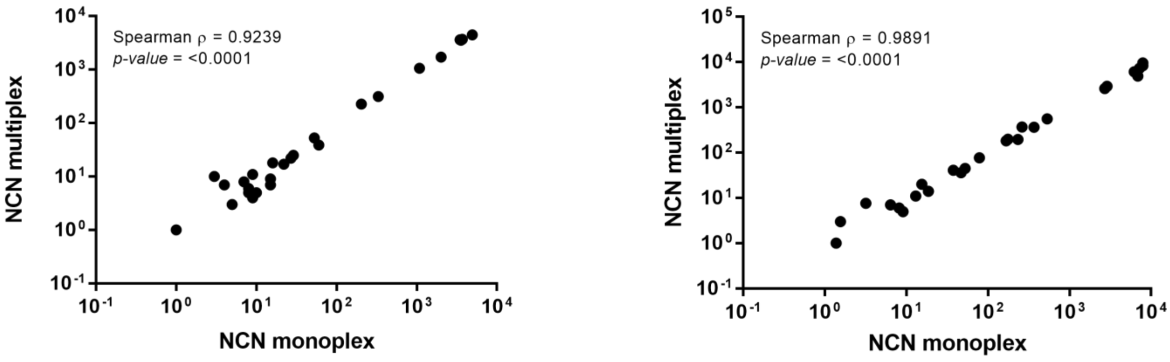

(**a**) (**b**)

Figure S1. Comparison of monoplex and multiplex dPCR assay for MDD/MRD quantification. Concordance of NCN *NPM-ALK* measured by monoplex and multiplex assays in 26 BM (**a**) and 26 paired PB (**b**) samples.

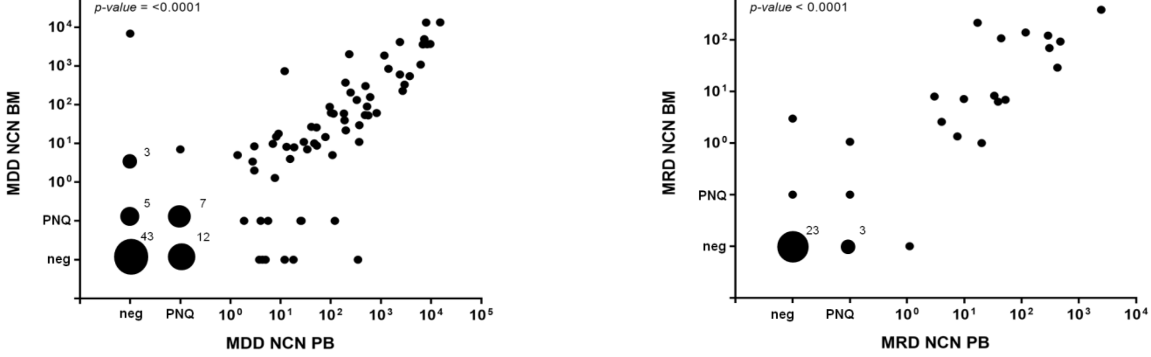

(**a**) (**b**)

Figure S2. Comparison of MDD and MRD quantification in BM and PB. Correlation of MDD (**a**) and MRD (**b**) results as NCN *NPM-ALK* measured in paired BM and PB samples from 138 and 47 patients, respectively.

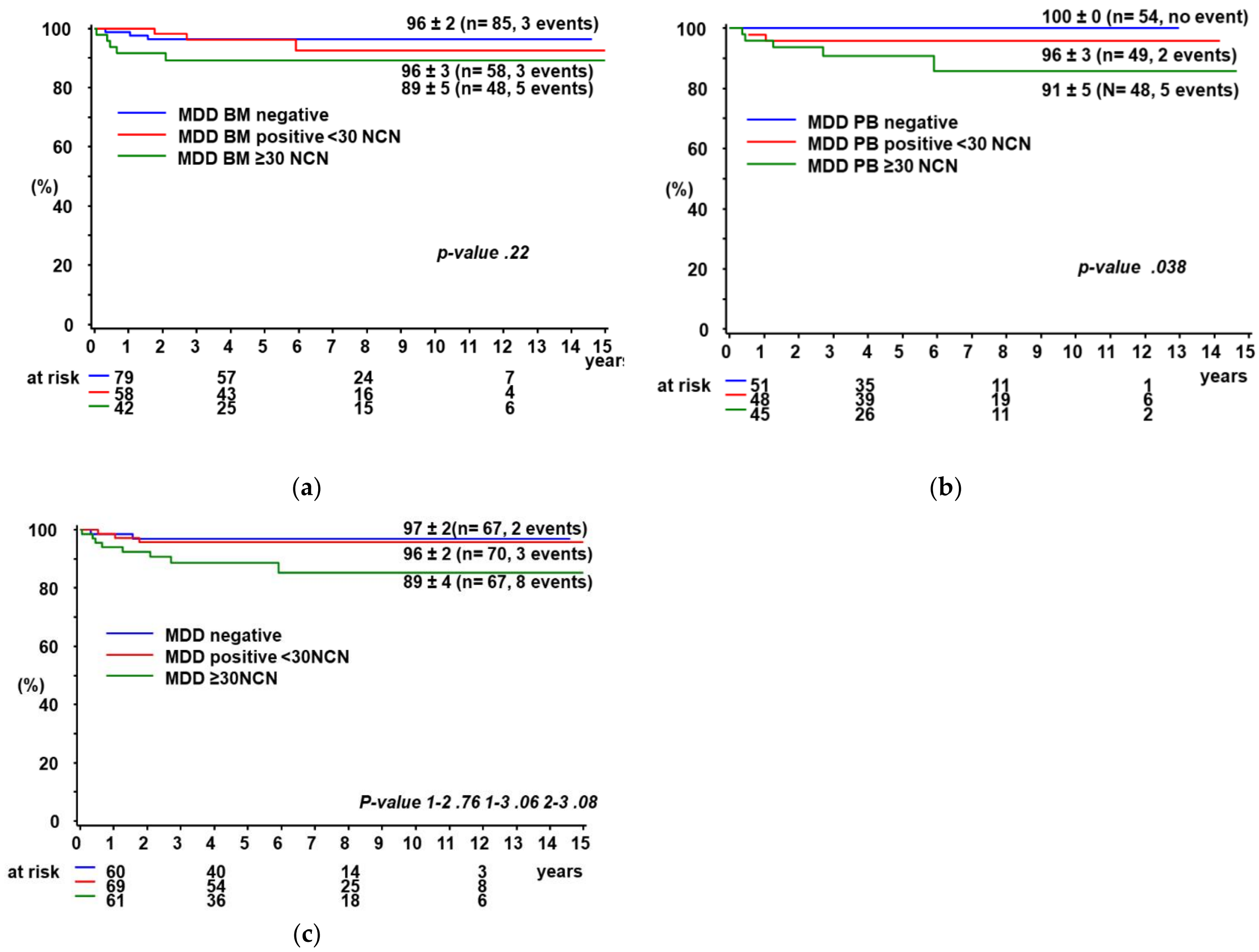

Figure S3. 5-year OS% according to MDD results (negative, positive < 30 NCN or ≥ 30 NCN) in BM (**a**), PB (**b**), combined (**c**).

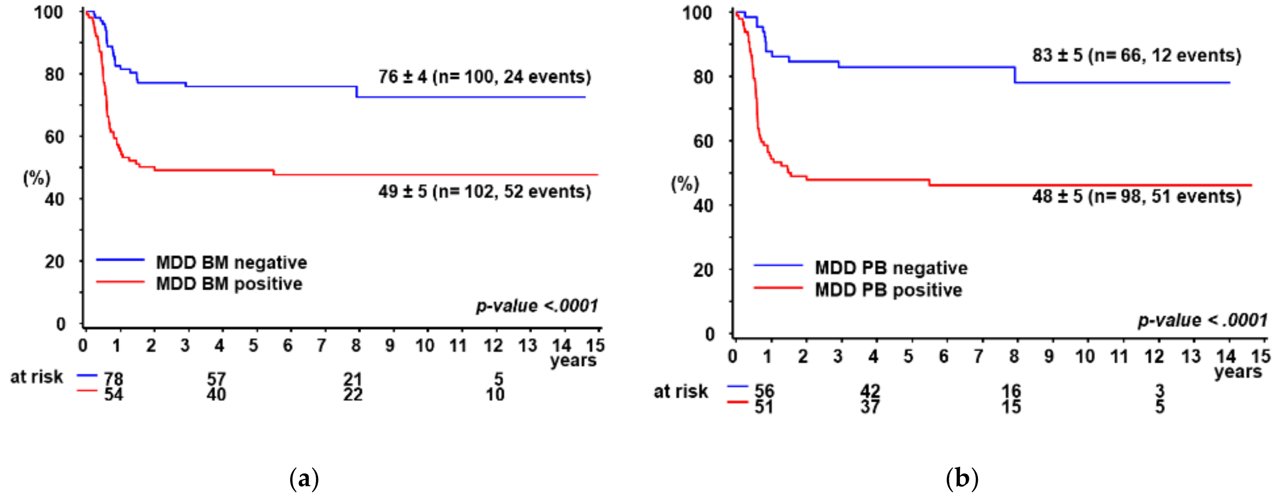

Figure S4. 5-year PFS% according to qualitative MDD results (negative or positive) in BM (a) or PB (b).

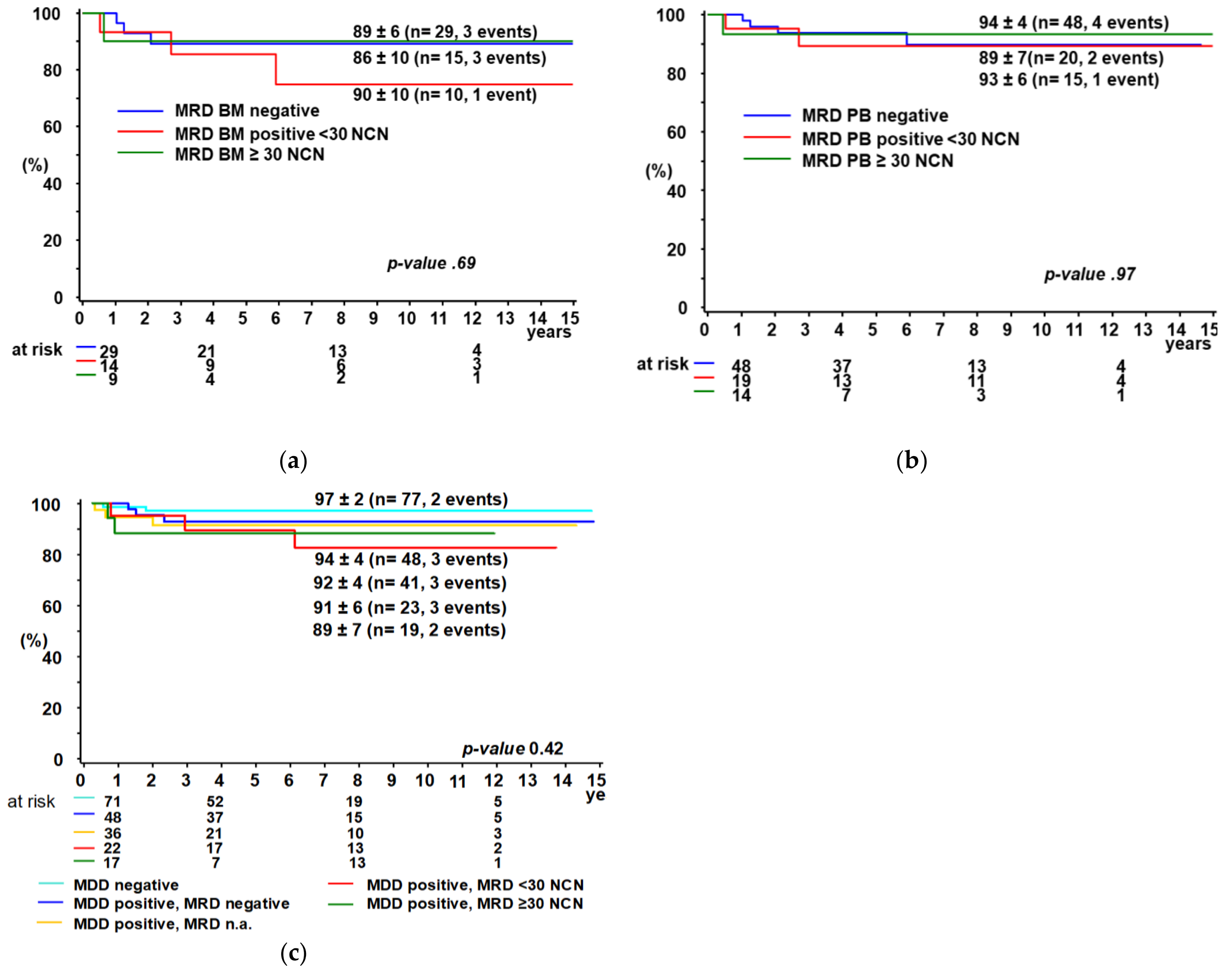

Figure S5. 5-year OS% according to MRD results (negative, positive < 30 NCN or ≥ 30 NCN) in BM (**a**), PB (**b**) and combined (**c**).

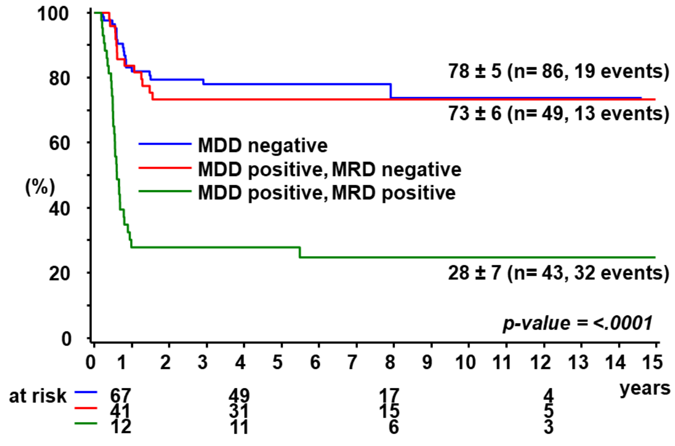

Figure S6. 5-year PFS% according to qualitative MDD and MRD results (negative or positive) in BM and/or PB.

Supplementary Table S1. MDD results in paired BM and PB samples or one of them, if only one was available.

**MDD N=204**	*MDD PB negative*	*MDD PB < 30 NCN*	*MDD PB ≥ 30 NCN*	*MDD PB n.a.*
MDD BM negative	43	19	1	22
MDD BM < 30 NCN	8	25	11	14
MDD BM ≥ 30 NCN	1	1	29	17
MDD BM n.a.	2	4	7	-

Supplementary Table S2. MRD results in paired BM and PB samples or one of them, if only one was available.

**MRD N=90**	*MRD PB negative*	*MRD PB < 30 NCN*	*MRD PB ≥ 30 NCN*	*MRD PB n.a.*
MRD BM negative	23	4	0	2
MRD BM < 30 NCN	2	7	4	2
MRD BM ≥ 30 NCN	0	1	6	3
MRD BM n.a.	23	8	5	-
